# IgE level in newborn umbilical cord and its relationship with some maternal factors

**DOI:** 10.1186/s12948-019-0115-1

**Published:** 2019-07-26

**Authors:** Iraj Mohammadzadeh, Mohsen Haghshenas, Samaneh Asefi, Reza Alizadeh-Navaei

**Affiliations:** 1 Non-Communicable Pediatric Diseases Research Center, Health Research Institute, Babol University of Medical Sciences, Imam Khomeini Street, Babol, 4731-741151 Iran; 2 Gastrointestinal Cancer Research Center, Mazandaran University of Medical Sciences, Sari, 48166-33131 Iran

**Keywords:** Allergy, IgE, Newborns

## Abstract

**Background and objectives:**

Allergic diseases are among major pediatric issues as they are highly prevalent and chronic. Therefore, identification of factors contributing to allergic disease could play a significant role in prevention of these conditions. This study aimed at investigating the IgE level in newborn’s umbilical cord blood and its relationship with some maternal factors.

**Methods:**

A cross-sectional study was conducted in 101 mothers and their newborns in Babol, Iran 2016. The samples were selected using non-probability convenience sampling. Information including newborn sex, gravidity, history of allergy before and during pregnancy (asthma, allergic rhinitis, eczema, hives, food allergy, and drug allergy), family history of allergy among mothers, history of exposure to secondhand smoke and pets, and delivery techniques was recorded. The IgE levels in newborn umbilical cord blood and maternal serum were measured using an IgE kit and ELISA technique.

**Results:**

The newborns included 53 females (52.5%) and 29 mothers had vaginal birth (28.7%). History of exposure to secondhand smoke was found in 15 samples (14.9%), and 18 participants reported exposure to pets (17.8%). The median IgE levels in newborns and their mothers were 0.41 and 98.6, respectively. In general, IgE level in all newborns was within the normal range, but, it was higher than normal in 15 mothers (14.9%). The IgE level was significantly higher in male newborns than that of the female newborns (p = 0.011). There were no significant differences in the IgE levels of mothers and their newborns on the basis of delivery technique and history of exposure to pets (p > 0.05).

**Conclusion:**

In this study, the IgE level in all newborns was within the normal range, and sex was found to be an effective factor in IgE levels.

## Background

Allergic diseases are a common problem with an increasing prevalence over the past decades, and a prevalent illness among children in Iran [1, 2]. There are several reports on increasing prevalence and severity of its side effects worldwide [3, 4]. Atopic diseases develop in people with a genetic background. However, some environmental factors, such as pediatric infectious diseases, exposure to allergens, socioeconomic status of families, and lifestyle play a pivotal role [5]. Accordingly, there is a growing interest in initial prevention of atopic diseases. Early diagnosis of vulnerable groups during prenatal period is the first essential step towards prevention and provision of a better treatment [6]. It seems that IgE in cord blood is a product of the fetus by the 11th week of gestation, which does not typically cross the placental barrier [7]. The role of umbilical cord blood IgE in prediction of atopic propensity was first proposed in a few decades ago as a valuable measure to identify newborns at high risk of atopy in future [8, 9]. The antepartum period has a vital role in evolution of the immune system which, in turn, has a long-run effect on health throughout the life span, therefore, fetal exposure to the intrauterine allergens is thought to be a significant factor in this regard [10]. Due to following reasons, the identification of effective factors may have a significant role in prevention of allergic diseases:

i. high prevalence and chronicity of allergic diseases have made them major challenges in today’s healthcare systems
ii. there are differences between different regions regarding the level of allergens, specifically those that cross the placental barrier.

This study aimed at investigating the IgE level in umbilical cord blood of a population of newborns in Babol, Iran to specify the relationship between this level and some maternal variables.

## Methods

This cross-sectional study was conducted in the mothers and their newborns, attending Babol Rouhani Hospital for giving birth during the fall and winter of 2016–2017. The participants were selected using non-probability convenience sampling. The minimum sample size estimation was approximately 100 patients based on p = 0.6, a = 0.05, and d = 0.1. Data was collected by recording the following information: demographic information (newborn sex, gravidity, season of birth, etc.), history of allergy before and during the pregnancy (asthma, allergic rhinitis, atopic dermatitis, urticaria, food allergy, and drug allergy), mother with a family history of allergy, history of exposure to secondhand smoke and pets, and delivery method. After obtaining the informed consent from parents, 1 cc of maternal serum and newborn umbilical cord blood samples were taken. The sampling was done using a 2 cc syringe. The samples were stored at 2–8 °C and then delivered to the laboratory for measurement of the total IgE. The IgE level in umbilical cord blood samples was examined using an IgE kit and nephelometry technique. High IgE level suggested that the mother and newborn blood cells were mixed. Therefore, cases with high IgE levels (n = 3) were excluded. The IgE levels in newborn umbilical cord blood and mother serum were measured using an IgE kit and ELISA technique. Based on kit handbook, IgE less than 13, 81 and 127 (IU/ml) for age less than 1, 1–7 years and more than 8 years, respectively, was considered as normal level. Parents provided informed consent for umbilical cord blood sampling. Data were analyzed in SPSS16 and Mann–Whitney U test was applied to compare the IgE level in mothers and their newborns based on different variables. In this study, the significance level was set at lower than 0.05. The one sample Kolmogorov–Smirnov test was used to investigate normal distribution of IgE level.

## Results

This study was conducted in 101 mothers and 101 newborns. The mean age of mothers was 30.7 ± 5.2 (17–41 years old). The mean maternal age of the participants was 38.4 ± 0.9 years old. Data about the distribution of sex, gravidity, and delivery technique is presented in Table 1. Data shows that 53 newborns were female (52.5%) and 29 mothers had vaginal birth (28.7%).

**Table 1:** The distribution of IgE level in mothers and their newborns based on some related factors

IgE	Median	Interquartile range	Mean	SD	p-value
Sex					
Mother					
Female	43.8	86.9	95.5	113.7	0.286
Male	38	87.6	102	161	
Newborn					
Female	0.3	0.2	0.31	0.22	0.011
Male	0.4	0.45	0.53	0.53	
Delivery					
Mother					
NVD	49	61.7	81.3	120.4	0.394
Cesarean section	38.9	106.2	105.5	144.1	
Newborn					
NVD	0.3	0.3	0.36	0.2	0.937
Cesarean section	0.35	0.3	0.43	0.47	
Passive smoking					
Mother					
No	40.5	81.37	92.1	128.9	0.717
Yes	87.1	113.8	135.6	180.7	
Newborn					
No	0.4	0.3	0.44	0.42	0.014
Yes	0.2	0.4	0.25	0.31	
Pet exposure					
Mother					
No	41.8	88.7	99.6	142.3	0.915
Yes	39.4	114.9	94	116.8	
Newborn					
No	0.3	0.3	0.38	0.33	0.591
Yes	0.3	0.35	0.57	0.67	

There were three mothers with allergic problems during labor (3%), one had a history of allergy (1%), and one mother was found with a family history of allergy (1%). Nine people had a history of underlying diseases (8.9%), including blood pressure disorder (n = 1), diabetes (n = 2), diabetes with blood pressure (n = 2), hypothyroidism (n = 2), and sarcoidosis (n = 2). There was a history of exposure to secondhand smoke in 15 participants (14.9%), and 18 reported exposure to pets (17.8%).

The median IgE levels (IU/ml) in newborns and their mothers were 0.41 and 98.6, respectively. In general, IgE level of all newborns was within the normal range; whereas, it was higher than normal level in 15 mothers (14.9%).

The distribution of IgE level in mothers and their newborns is presented in Table 1, based on the newborn sex, delivery method, and exposure to secondhand smoke and pets during the pregnancy. The IgE level in male newborns was found to be significantly higher than that in female newborns (p = 0.011). In addition, the IgE level in newborns born to mothers with a history of prenatal exposure to secondhand smoke was significantly lower (p = 0.014). There was no any significant relationship between other cases.

## Discussion

In present study, the IgE level of all newborns was within a normal range, but it was higher than normal range in 15 mothers (14.9%). In an investigation of 763 childbirths, including 724 term births and 39 preterm births 14% of mothers had high levels of IgE [11]. This is consistent with current results. But, Hernandez et al. reported high levels of IgE in umbilical cord blood (53.7%) [12]. Studies have shown that the IgE levels in mother and newborns may have a role in future development of allergic diseases. A cohort study showed that the risk of allergy development at the age of 4 (OR = 2.92) and 10 (OR = 1.73) was significantly correlated with increased IgE level in newborn umbilical cord blood [13]. Hence, neonatal IgE level is believed to be correlated with allergic diseases, specifically in early childhood.

In current study, significantly higher levels of IgE were seen in male newborns (p = 0.011). Consistent with our findings, Petrovičová et al. also found that the IgE level in umbilical cord blood was higher in male newborns [14], so, the sex is an important factor concerning the IgE level [11]. In contrast, some reported no significant relationship between the sex and umbilical cord blood IgE [15]. Higher levels of IgE in male newborns might be related to higher prevalence of atopy and allergic diseases in boys [16].

In current study, delivery method did not significantly influence the IgE levels of mothers and their newborns. But Nabavi et al. showed a relationship between the umbilical cord blood IgE and delivery method [7]. Similarly, Petrovičová et al. reported that the IgE level was higher in newborns born by cesarean section than those born via vaginal birth. These different rates might be due to low number of samples and vaginal births in our study.

The IgE level was found to be significantly lower in newborns of 15 mothers (14.9%) with a history of exposure to secondhand smoke (p = 0.014). In contrast, Gurbuz et al. showed no significant correlation between exposure to secondhand smoke and umbilical cord IgE level [17].

In the current study, the IgE levels of mothers and their newborns was not significantly different based on the childbirth method Two studies investigating the relationship between prenatal exposure to pets and IgE level in newborn umbilical cord revealed different results. Hernandez et al. showed that having pets could influence the umbilical cord IgE level [12], but Gurbuz et al. reported no significant relationship between exposure to pets and IgE level in newborn umbilical cord [17]. The latter finding is consistent with present results [12].

This study was associated with some limitations, including lack of a control group. The researchers suggest further studies with greater sample size, as of longitudinal studies into the effect of IgE level in newborns with allergic diseases during childhood.

## Conclusion

Results showed that the IgE level in all newborns was within the normal range, but sex was found to be an effective factor.

## Data Availability

The datasets analysed during the current study are available from the corresponding author on reasonable request.

## References

[1] Mohammadzadeh I, Ghafari J, Savadkoohi RB, Tamaddoni A, Esmaeili Dooki MR, Alizadeh-Navaei R (2008). The prevalence of asthma, allergic rhinitis and eczema in North of Iran: the International Study of Asthma and Allergies in Childhood (ISAAC). Iran J Pediatr..

[2] Viegi G, Baldacci S, Vellutini M, Carrozzi L, Modena P, Pedreschi M, Maggiorelli F, Di Pede F, Paoletti P, Giuntini C (1994). Prevalence rates of diagnosis of asthma in general population samples of northern and central Italy. Monadei Arch Chest Dis..

[3] Gergen PJ, Mullally DI, Evans R (1988). National survey of prevalence of asthma among children in the United States, 1976 to 1980. Pediatrics.

[4] Clifford RD, Radford M, Howell JB, Holgate ST (1989). Prevalence of respiratory symptoms among 7 and 11 year old schoolchildren and association with asthma. Arch Dis Child.

[5] Ayatollahi SMT, Ghaem H (2004). Prevalence of Atopic diseases (Allergic rhinitis, Urticaria, Eczema) and its correlations in primary school children, Shiraz, Iran. J Gorgan Uni Med Sci..

[6] Brown MA, Halonen MJ, Martinez FD (1997). Cutting the cord: is birth already too late for primary prevention of allergy?. Clin Exp Allergy.

[7] Nabavi M, Ghorbani R, Asadi AM, Faranoush M (2013). Factors associated with cord blood IgE levels. Asian Pac J Allergy Immunol.

[8] Kjellman NI, Croner S (1984). Cord blood IgE determination for allergy prediction—a follow-up to seven years of age in 1,651 children. Ann Allergy..

[9] Bousquet J, Menardo JL, Viala JL, Michel FB (1983). Predictive value of cord serum IgE determination in the development of “early-onset” atopy. Ann Allergy..

[10] Prescott SL (2003). Early origins of allergic disease: a review of processes and influences during early immune development. Curr Opin Allergy Clin Immunol.

[11] De Amici M, Perotti F, Marseglia GL, Ierullo AM, Bollani L, Decembrino L, Licari A, Quaglini S, Stronati M, Spinillo A (2017). Cord and blood levels of newborn IgE: correlation, role and influence of maternal IgE. Immunobiology.

[12] Hernandez E, Barraza-Villarreal A, Escamilla-Nunez MC, Hernandez-Cadena L, Sly PD, Neufeld LM, Romieu I (2013). Prenatal determinants of cord blood total immunoglobulin E levels in Mexican newborns. Allergy Asthma Proc..

[13] Sadeghnejad A, Karmaus W, Davi S, Kurukulaaratchy RJ, Matthews S, Arshad SH (2004). Raised cord serum immunoglobulin E increases the risk of allergic sensitisation at ages 4 and 10 and asthma at age 10. Thorax.

[14] Petrovičová O, Bánovčin P, Babušíková E, Jeseňák M (2016). Factors modifying cord blood IgE levels—a pilot study. Epidemiol Mikrobiol Imunol.

[15] Karimi M, Mojibian M, Nodoshan H, Bidaki R, Kholacezadeh G, Rafiee P, Saberi Z (2012). Relationship between stress during pregnancy and cord blood IgE level. J Shahid Sadoughi Univ Med Sci Yazd.

[16] Tu YL, Chang SW, Tsai HJ, Chen LC, Lee WI, Hua MC, Cheng JH, Ou LS, Yeh KW, Huang JL, Yao TC (2013). Total serum IgE in a population-based study of Asian children in Taiwan: reference value and significance in the diagnosis of allergy. PLoS One..

[17] Gurbuz T, Karakol B, Onal ZE, Tabak Y, Nuhoglu C, Ceran O (2015). Evaluation of in utero sensitization by screening antigen-speciic immunoglobulin E levels in umbilical cord blood.. Postepy Dermatol Alergol..

